# Impact of Fiscal Policy for Sugar-Sweetened Beverages on Reducing the Burden of Disease and Healthcare Costs in Brazil: A Simulation Study

**DOI:** 10.3390/nu18030435

**Published:** 2026-01-28

**Authors:** Luciana Bertoldi Nucci, Ben Amies-Cull, Flavia Mori Sarti, Wolney Lisboa Conde, Carla Cristina Enes

**Affiliations:** 1 Postgraduate Program in Health Sciences, School of Life Sciences, Pontifical Catholic University of Campinas (PUC-Campinas), Campinas 13060-904, SP, Brazil; cacenes@gmail.com; 2 Nuffield Department of Primary Care Health Sciences, University of Oxford, Oxford OX2 6GG, UK; ben.amies-cull@phc.ox.ac.uk; 3 School of Arts, Sciences and Humanities (EACH-USP), University of São Paulo, São Paulo 03828-000, SP, Brazil; flamori@usp.br; 4 Department of Nutrition, School of Public Health, University of São Paulo, São Paulo 01246-904, SP, Brazil; wolney@usp.br

**Keywords:** tax policies, sugar-sweetened beverages, non-communicable disease, modeling, economic modeling, health policy, Brazil

## Abstract

**Background/Objectives**: Sugar-sweetened beverage (SSB) consumption has been linked to obesity, metabolic diseases, and rising healthcare costs. This study aimed to assess the impact of a 20% excise tax on SSBs in Brazil on obesity/overweight prevalence, seven musculoskeletal and cardiovascular diseases, and related healthcare costs, with their associated impacts on health inequalities. **Methods**: Using 2017/2018 Brazilian Household Budget Survey data for baseline consumption and own- and cross-price elasticities for taxed beverages, we estimated changes in caloric consumption for the entire population and for lower- and upper-income quartiles. The PRIMEtime dynamic individual-level simulation model projected body weight changes, lifetime Quality-Adjusted Life-Years (QALYs), healthcare costs (discounted at 5%), and disease cases (20-year horizon). **Results**: A 20% excise SSB tax was projected to reduce obesity prevalence by 1.7 percentage points in men and 1.5 percentage points in women, from baseline rates of 19.8% and 23.6%, respectively. Lifetime gains were estimated at 17,878 QALYs per million men and 12,181 per million women, alongside healthcare cost savings of Int$520 million. Impacts varied by income, with smaller health gains in the lowest quartile and higher among the wealthiest. Over 20 years, the tax could avert 1784 cases of type 2 diabetes mellitus/100,000 adults (52% in men) and 1070 cases of ischemic heart disease/100,000 adults (80% in men). **Conclusions**: A 20% excise SSB tax in Brazil could yield large health and cost benefits. With the recent approval of the Selective Tax under Complementary Law 214/2025, Brazil has a timely opportunity to translate these projected benefits into effective public health policy.

## 1. Introduction

The intake of sugar-sweetened beverages (SSBs) has been linked to higher calorie consumption, weight gain, and the onset of several chronic conditions, including type-2 diabetes (T2DM), other metabolic disorders, and cancer [1,2,3]. In addition, previous research has shown that the long-term consumption of SSBs is associated with increased mortality, mainly due to cardiovascular disease [4], as well as the considerable economic burden associated with obesity-related complications [5,6].

The taxation of sugar-sweetened beverages emerged as a promising fiscal policy intervention with measurable potential to reduce the burden of disease, prevent avoidable deaths, and reduce healthcare costs in Brazil [7,8,9,10]. The World Health Organization (WHO) has recommended implementing tax policies to reduce the consumption of sweetened beverages, improve diets, and reduce chronic diseases [11]. In response, governments worldwide have introduced structural policies to guide healthier consumer choices [1,12].

Latin America has taken a leading role in adopting taxes on SSBs [13]. In 2014, Mexico introduced a 10% tax on SSBs, which led to a 9.7% decline in consumption within two years [14]. Projections over 10 years suggested that this policy could result in a 2.5% reduction in adult obesity prevalence [15]. In 2016, Chile adopted a comprehensive strategy to curb SSB consumption, which included increasing the existing tax from 13% to 18%. By the following year, consumption of taxed beverages had fallen by 23.4%, accompanied by a 27.5% reduction in caloric intake from these drinks. Overall, fiscal and regulatory measures targeting SSBs in Latin America have successfully decreased consumption and are projected to yield significant public health gains [16,17].

In Brazil, according to the Household Budget Survey (POF 2017–2018), foods high in sugar consumed by the Brazilian population include soft drinks, processed juices, sweetened dairy beverages, sweet biscuits (filled and unfilled), cakes, chocolates, sweets in general (jams, condensed milk), ice cream, as well as coffees and juices with added sugar and ultra-processed products. On average, adults in Brazil consume almost 62 L of sugary drinks per year, with soft drinks representing 65% of that volume [18].

In 2024, Brazil took a significant step toward promoting healthier food environments by approving a tax reform that included SSBs among the products subject to higher taxation. Considering the Brazilian tax system, it was proposed that a Contribution for Intervention in the Economic Domain (CIDE) be created for sweetened beverages. One of its advantages is that the CIDE allows the revenue it generates to be earmarked for specific funds, programs, and actions, thereby guaranteeing benefits to the population. This policy aligns with growing global efforts to reduce the consumption of unhealthy foods and address the rising attributable burden of disease. Simulation studies conducted in Brazil have projected substantial health and economic benefits from taxing SSBs [7,8,9,10]. One national modeling study estimated that a 20% excise SSB tax could prevent approximately 2.8 million cases of obesity over 10 years, while a 30% tax could avert over 3.8 million cases [10]. The same study also projected that such a measure could prevent tens of thousands of new cases of diabetes, cardiovascular diseases, and obesity-related cancers, along with thousands of premature deaths. Additionally, the tax would yield significant savings for the Brazilian Unified Health System (SUS), thereby reducing healthcare costs associated with the treatment of diet-related chronic conditions [10].

Although previous studies in Brazil have evaluated the potential effects of SSB taxation on health outcomes, mortality, and healthcare expenditure, there is still limited evidence on how these impacts may vary across different income groups. Given that low-income individuals may be simultaneously more sensitive to price changes and have a greater exposure to diet-related risks, investigating the differential effects of fiscal policies by income level is essential to informing equitable and effective public health strategies. It has previously been noted that despite a growing body of modeling studies on SSB taxes, equity analyses are lacking [19].

Thus, this study aimed to assess the potential impact of a 20% excise SSB tax on the prevalence of obesity/overweight, the burden of T2DM, cardiovascular disease, chronic back pain, hip/knee osteoarthritis, and cost savings for individuals in Brazil across different income groups.

## 2. Materials and Methods

### 2.1. Model Overview

**Figure 1:**
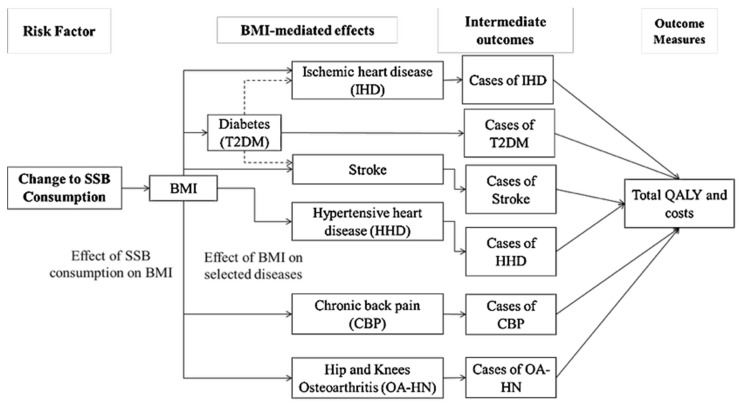
Representation of the PRIMEtime model diagram of a simulated 20% excise SSB tax for the intervention population.

This study employed the PRIMEtime model, a proportional multistate life table model, to simulate the potential health and cost impacts of a 20% excise SSB tax in Brazil. The methods are described in detail elsewhere [20,21], and were adapted for use in Brazil in the present study (Figure 1). Here, we estimated the policy’s impacts on ischemic heart disease, stroke, hypertensive heart disease, T2DM, low back pain, osteoarthritis of the hip, and osteoarthritis of the knee, including their associated Quality-Adjusted Life-Year (QALY) and healthcare cost impacts. Briefly, PRIMEtime consists of a linked multistate life table model that divides the population into three states for each disease outcome: healthy, diseased, and dead (or two states, excluding death for chronic back pain and osteoarthritis). State transition parameters determining the flow between states were estimated from published epidemiological data from the Global Burden of Disease (GBD) study [22], with secondary modeling applied using the Disbayes model [23] to produce consistent sets of incidence and case fatality rates, along with associated prevalence (Supplementary File Figures S1–S7). The model is structured at the population level and follows the Markovian assumption, meaning that individuals are not identified during the simulation process, and transitions between states are independent of the duration spent in each state.

Scenario definition: An intervention (counterfactual) scenario with a 20% excise SSB tax was modeled. We assumed a 100% pass-on rate, representing a best-case scenario of full tax transmission to consumer prices, which was compared against the baseline scenario of no SSB tax, considering that the current Brazilian tax reform (Complementary Law No. 214/2025) is based on a dual value-added tax system (Tax on Goods and Services corresponding to 17.7%, IBS; and Contribution on Goods and Services corresponding to 8.8%, CBS), in addition to the selective excise tax (Imposto Seletivo, IS) on specific products (including SSBs), which would be effective from 2027. However, the IS aliquot is still under evaluation; therefore, based on estimates for the IBS and CBS, which will substitute part of the current consumption taxes in Brazil, the present study evaluated a 20% aliquot incorporated into SSB prices. It was assumed that due to higher prices, demand and consumption of SSBs will be suppressed, and that the overall energy intake is lower as a consequence, consistent with trial evidence that SSB consumption leads to weight gain [24]. The baseline starting year was set to 2022, with a closed cohort based on the 2022 Brazilian Census population [25], and the intervention effect occurring from age 20; no new individuals entered the cohort over time. QALYs and healthcare costs were estimated over the remaining lifetime of the cohort, both discounted to 5%, while disease cases were counted over 20 years (undiscounted). The intervention was assumed to remain in place for the lifetime of the cohort, with its estimated effects persisting over the entire simulation period. The main parameters used in the model to estimate the impact on Body Mass Index (BMI) were the tax pass-on rate, cross- and own-price elasticities, total energy intake, and baseline BMI. All model parameters are described in the Supplementary Materials (Supplementary File Table S1).

### 2.2. Data Source and Assumptions

#### 2.2.1. Cross- and Own-Price Elasticities

We used both the own- and cross-price elasticities of demand, taken from a previous study [26], to measure changes in consumption in response to changes in SSB prices. The own- and cross-price elasticities represent the proportional change in the purchase of a product in response to a 1% increase in its price and the price of other products, respectively. The price elasticities (own- and cross-price elasticities) for SSBs adopted in this study were measured for non-alcoholic beverages, using the Quadratic Almost Ideal Demand System (QUAIDS) model [27] and estimated by Dassow et al. [26] for the total population and for those in the lower- and upper-income quartiles. Briefly, the demand for non-alcoholic beverages was estimated using data from the 2017–2018 Household Budget Survey (POF), a Brazilian cross-sectional survey of household expenditure [18] covering fourteen categories of beverages. For this study, we applied the elasticities that reached significance (*p* < 0.05) for seven products for the simulation of taxation on consumption: (1) sugary juices/drinks, (2) soft drinks, (3) sugary dairy drinks, (4) sweetened juices/drinks, (5) sweetened dairy drinks, (6) light/diet soft drinks, and (7) sports drinks/energy drinks (Supplementary File Table S2).

#### 2.2.2. Baseline BMI, Beverage Consumption, and Total Energy Intake

The baseline BMI was calculated as self-reported weight (in kilograms) divided by height (in meters) squared, using data from the 2019 National Health Survey (Pesquisa Nacional de Saúde, PNS) [28]. The PNS is a population-based household survey with a cross-sectional design, representative of individuals aged 15 years or older residing in private households across Brazil. Among the surveyed population, data from 85,825 individuals aged 20 years or older were used to calculate the BMI. The survey employed a complex sampling strategy, utilizing a three-stage cluster sampling plan. Mean BMI calculations for age groups were performed using PROC SURVEYMEANS in SAS 3.82 (Enterprise Edition), which incorporates survey weights, strata, and primary sampling units (PSUs). Design-based standard errors were calculated using the Taylor series linearization method to account for complex design effects, including clustering (Supplementary File Table S3).

The baseline consumption of SSBs and the total daily energy intake of Brazilian adults were estimated from 24-h dietary recalls that included all food consumed both inside and outside the home from the POF 2017–2018 [18]. Participants reported their beverage intake in standard servings, which were then converted into milliliters with a factor of 250 mL/serving. Average intake volumes and total energy intake were calculated across the full sample and for the lower- and upper-income groups, disaggregated by sex and age, using survey sampling weights in all estimates (Supplementary File Tables S4 and S5).

### 2.3. Changes in Energy Intake, Body Weight, BMI, and Overweight and Obesity Prevalence

We considered the own- and cross-price elasticities of SSBs by income level to estimate the impact of the tax on total energy intake. The changes in weight and BMI resulting from the reduction in calorie intake were estimated using a microsimulation approach implemented in the ‘bw’ package in R, which implements the dynamic body weight change equations proposed by Hall et al. (2011) [29]. This model has been frequently used to estimate the potential impact of taxing SSBs on weight [15,30,31]. It considers changes in extracellular fluid, glycogen, and fat and lean tissue resulting from alterations in caloric intake. The model assumes that a reduction of 100 kJ per day results in a reduction of one kilogram of body weight, with half of the weight change achieved in approximately one year and 95% of the weight change achieved in about three years. In addition, it was assumed that the same level of individual physical activity was maintained.

### 2.4. Direct Medical Costs for Selected Noncommunicable Diseases (NCDs)

The NCDs included in this study were selected based on the GBD 2021 [22], with a focus on high body-mass index (BMI) as a risk factor and prioritizing conditions with the highest burden in Brazil. The following diseases were selected for the analysis: ischemic heart disease (IHD), T2DM, stroke, and hypertensive heart disease (HHD). These four conditions account for approximately 65% of NCD deaths attributable to high body mass index in Brazil. Additionally, when including the three other conditions that contribute most to Disability-Adjusted Life Years (DALYs)—osteoarthritis of the knee, osteoarthritis of the hip, and low back pain—the seven diseases together reflect approximately 75% of the total DALY burden attributable to high BMI in the country [22].

Cost data for the analysis were obtained from the Brazilian Public Hospitals Information System (SIH) of the SUS for each selected disease in 2021 [32]. The number of hospitalizations and total costs were extracted, and the mean and standard deviation were calculated by sex for each age group based on primary and secondary International Classification of Diseases (ICD) codes for admissions (Supplementary File Table S6). Costs were initially extracted in Brazilian Reais (BRL) and subsequently converted to international dollars ($) using the 2021 purchasing power parity (PPP) conversion factor of 2.531, obtained from the World Development Indicators of the World Bank. This adjustment enables more accurate comparisons with findings from studies in diverse countries and different periods. The number of patients admitted per year was calculated as the proportion of cases reported in the SUS data by 5-year age group and sex, and total cases were obtained from the GBD data. Total cases were the prevalence for low back pain and T2DM, and the incidence for IHD, stroke (due to being associated with acute treatment events), and osteoarthritis of the hip and knee (with the majority of lifetime costs accruing at a single admission, i.e., joint replacements). Costs for HHD were not calculable due to inconsistencies between the SUS and GBD data, so they were assumed to be zero.

### 2.5. Chronic Diseases Impact Modeling

Data on the number of deaths, prevalent cases, and incident cases for selected diseases were extracted from the GBD 2021 [22]. A smoothing process was applied to these data to calculate case-fatality rates for fatal diseases (IHD, stroke, T2DM, HHD). This process involved generating estimates of the number of deaths, prevalent cases, and incident cases for each selected disease by age and sex, which were then used for subsequent simulations and calculations of case fatality rates. For HHD, since incident case data were unavailable in the GBD 2021 [22], estimates for this parameter were obtained using the ‘disbayes’ package in R.

The smoothing process and subsequent analyses using ‘disbayes’ yielded refined estimates of deaths, prevalence, incidence, and case fatality rates for each selected disease (T2DM, IHD, HHD, and stroke) by year of age and sex. The ‘disbayes’ package employs a Bayesian three-state life table model to estimate epidemiological indicators for chronic diseases with incomplete or aggregated data, leveraging the logical dependencies between the disease states. Disease remission was assumed to be zero [23].

PRIMEtime was used to simulate the impact of changes in total caloric intake and, consequently, BMI, on the aforementioned range of BMI-related chronic diseases. The PRIMEtime model operates by simulating changes in obesity prevalence attributable to the intervention. Relative risks associated with high BMI for each condition were extracted from the GBD 2019 [33] and used in the model to estimate the impacts of changes to risk on disease incidence rates (Supplementary File Table S7).

Like other proportional multistate life table models, PRIMEtime assumes that the prevalence of one disease does not affect the risk of any other. An exception to this rule is diabetes, which operates both as a disease endpoint and as a risk factor for IHD and stroke in the model. To quantify cumulative parametric uncertainty throughout the modeling process, we conducted 2000 Monte Carlo runs in PRIMEtime. This analysis was used to estimate lower and upper uncertainty intervals (UIs) for cases of the seven BMI-related NCDs, as well as their associated QALY and healthcare cost impacts. Parameters with uncertainty applied were the BMI-disease relative risks, the diabetes-disease relative risks, utility decrements, healthcare costs, and intervention effects.

### 2.6. Sensitivity Analyses

We conducted sensitivity analyses to assess the robustness of our results in relation to key methodological assumptions by varying the discount rate applied to future health and cost outcomes. The base-case analysis used a 5% annual discount rate, consistent with Brazilian guidelines for economic evaluations in public health. In addition, we re-estimated all outcomes under two alternative scenarios to evaluate the impact of time preference on our projections: one with no discounting (0%) and another with a 10% per-year discount rate.

## 3. Results

A reduction in daily energy intake was observed across the population. The overall mean (95% confidence interval—95% CI) change in energy intake after a 20% excise SSB tax for the total population was−26.4 (−27.2; −25.5) kcal/person/day. When stratified by income level, the mean change was −18.1 (−19.4; −16.8) kcal/person/day among individuals in the lower income level and −29.0 (−30.5; −27.4) kcal/person/day among those in the upper income level. The analysis revealed that the tax had a greater effect on men than on women, particularly among individuals in the upper income levels (Table 1). Detailed data by age groups showed greater effects among the youngest of both genders, as described in Supplementary File Table S8.

Table 2 presents the prevalence of overweight and obesity before and after taxation for the total population, categorized by sex and age group. Overall, the estimated reductions among men were 0.94 percentage points (pp) for overweight and 1.68 pp for obesity. Among women, the reductions were slightly lower, at 0.56 pp for overweight and 1.53 pp for obesity.

The largest reductions in overweight prevalence were observed in individuals aged 85 years or over for both genders. Notable decreases were also observed among men in the 20–24 (−2, 8 pp) and 75–79 age groups. A 1.6 pp reduction was observed among women in the 35–39 age group, and a 1.1 pp decrease was observed in both the 20–24 and 60–64 age groups. Regarding obesity, a reduction of at least two pp was observed in men aged 45–49, 60–64, and 70–74, and in women aged 25–29 and 70–74.

**Figure 2:**
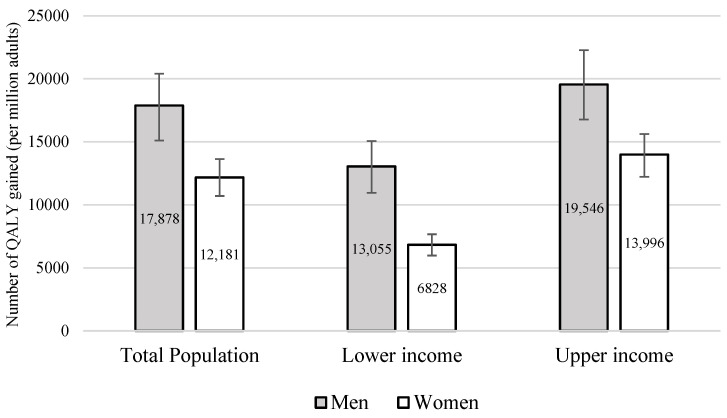
Estimated QALY gained (per million adults) over the lifetime, by sex and income levels, with 95% uncertainty intervals.

Figure 2 describes the estimated gains in QALYs for the modeled diseases. Over a lifetime, the tax is projected to generate 17,878 QALYs per million men and 12,181 QALYs per million women, with a lower impact at the lowest income level and greater benefits at the highest. For the Brazilian adult total population (148.5 million), this corresponds to 1,264,633 QALYs gained for men and 947,705 for women. When stratified by income level, the estimated gains were 230,939 QALYs for men and 132,841 for women in the lowest income level, and 345,658 for men and 272,204 for women in the highest income level.

**Figure 3:**
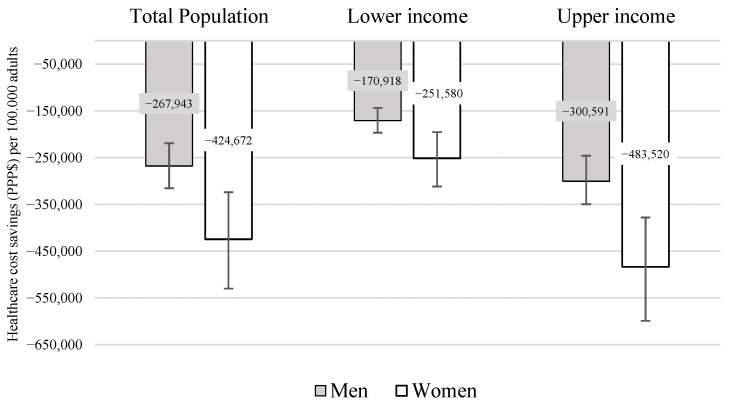
Estimated healthcare cost savings for modeled diseases over the lifetime ($PPP), per 100,000 adults, by sex and income levels, with 95% uncertainty intervals. Modeled diseases: Type 2 diabetes (T2DM), Ischemic heart disease (IHD), hypertensive heart disease (IHD), stroke, knee osteoarthritis, hip osteoarthritis, low back pain. PPP: purchasing power parity.

Figure 3 presents the projected healthcare cost savings for the seven modeled diseases following implementation of the 20% excise SSB tax. Savings were estimated at $267,943 per 100,000 men and $424,672 per 100,000 women, again with lower savings in the lowest income level and higher savings among the wealthiest. These per capita values were derived from the total savings estimates for the Brazilian adult population, amounting to $189.54 million for men and $330.39 million for women. By income level, savings reached $30.23 million for men and $48.94 million for women at the lowest income level, and $53.16 million for men and $94.04 million for women at the upper income level.

Table 3 presents the projected impact of a 20% excise SSB tax on the number of averted cases for selected diseases over 20 years. More than 650 thousand cases of T2DM would be averted in each sex, corresponding to 922 and 862 cases averted per 100,000 men and women, respectively. For IHD, the reduction was 4.1 times greater among men (858/100,000) than among women (212/100,000), whereas for HHD, the reduction was 4.6 times greater among women (401/100,000) than among men (87/100,000). Stroke cases would be reduced by approximately 120 per 100,000 individuals. Regarding musculoskeletal conditions, knee osteoarthritis would be reduced by 124 cases per 100,000 men and 206 cases per 100,000 women, while hip osteoarthritis would decrease by roughly 35 cases per 100,000 individuals. For low back pain, the reduction among women (1424/100,000) was 2.1 times greater than among men (672/100,000).

Sensitivity analysis using discount rates of 0% and 10% revealed substantial variation in both health and economic outcomes. The estimated QALY gained ranged from 82.9 to 6.9 for men and 74.1 to 3.8 for women in the total population, respectively, highlighting the strongest influence of discounting on long-term health benefits. Similarly, healthcare cost savings per adult varied markedly: at 0%, the model projected a reduction of $634,000 per 100,000 men and $913,000 per 100,000 women, while at 10%, the savings diminished to $156,000 per 100,000 men and $249,000 per 100,000 women across the total population. These patterns were also observed for both the lower- and upper-income levels (Table 4).

## 4. Discussion

We conducted a simulation study to evaluate the potential impact of a 20% excise SSB tax on the prevalence of overweight and obesity, the burden of selected NCDs, and cost savings for individuals across different income groups in Brazil. Our model predicted that a 20% excise SSB tax may reduce the prevalence of obesity by 1.6 pp for the total adult population, yielding approximately 14,894 additional QALYs per million adults and saving an estimated $350,033 (in 2021 PPP) per 100,000 adults in healthcare expenditure over a lifetime, related to the modeled conditions. Over a 20-year horizon, these results represent an expressive number of averted cases per 100,000 adults: 1784 for T2DM, 1070 for IHD, 488 for HHD, 239 for stroke, 331 and 69 for knee and hip osteoarthritis, respectively, and 2096 for low back pain.

The impact of reducing SSB consumption on overweight and obesity prevalence has been consistently reported across modeling studies. A recent systematic review of SSB taxes identified 61 articles of variable quality and reporting standards examining potential effects of SSB taxes, 25 of which modeled ad valorem taxes, and 15 of the 61 used life table modeling, as in this paper. It noted that equity analyses, such as those completed here, are needed to better inform policymakers with greater detail for decision making [19]. In Brazil, prior modeling by Basto-Abreu estimated a similar reduction of 1.6 pp in obesity prevalence using the same 20% excise tax on SSB [8]. Perelli et al. estimated that 2.7% of all overweight and obesity cases among adults were attributable to SSB consumption [34]. Our findings also align with the range observed internationally: a comparable 20% tax scenario was projected to reduce obesity by 1.74 pp in men and 1.21 pp in women in Germany [35], and by 1.34 pp in men and 0.56 pp in women in Mexico [36]. In Canada, modeled reductions varied by sex and income group: a 20% excise SSB tax was projected to reduce obesity prevalence by 1.33 pp among women in the lowest income quintile and to 1.18 pp among those in the highest, whereas for men, the reduction was slightly larger in the highest income group (1.89 pp) than in the lowest (1.73 pp) [37]. This mixed pattern underscores the importance of context- and sex-specific analyses when evaluating the equity implications of fiscal policies.

Beyond the reduction in overweight and obesity prevalence, the projected gain of approximately 2.21 million QALYs for the total Brazilian population underscores the substantial health benefit and likely high public sector perspective cost-effectiveness of implementing a 20% excise SSB tax in Brazil. While differences in modeling approaches, time horizons, population size, and discounting assumptions may account for variation in absolute figures, other studies consistently indicate that an SSB tax of this magnitude would yield meaningful improvements in population health. For instance, a modeling study in Germany evaluating a similar 20% ad valorem SSB tax projected a gain of 106,000 QALYs (using a 3% discount rate) over 20 years for the total population aged 30–90. That study considered BMI, stroke, coronary heart disease, and T2DM, and a pass-through to consumers of 82% [38]. Similarly, an economic evaluation in Canada estimated a total gain of 1.5 million QALYs (discounted at 1.5%) for the adult population (aged 20 and above) over a lifetime horizon, from a tax of CAD 0.015/oz on SSBs [39]. Furthermore, a quasi-experimental study evaluating the SSB tax implemented in Oakland on 1 July 2017 (at $0.01/oz), estimated QALY gains of 94.0 and 967.6 per 10,000 people over 10-year and lifetime horizons, respectively (3% discounting) [40]. The consistency of QALY gains observed across different international settings demonstrates that SSB taxation can yield health and quality-of-life benefits.

The economic benefits of the proposed SSB tax also project savings in direct healthcare expenditures. Our model predicts an average reduction of $163 (2021 PPP) per adult in healthcare costs associated with the selected NCDs, amounting to an estimated $12 billion (2021 PPP) in total savings for the public health system over the cohort’s lifetime (5% discounting). These projections are broadly consistent with other recent modeling studies. A 2024 national modeling study similarly projected that a 20% excise SSB tax in Brazil would reduce obesity-related healthcare costs by USD 13.3 billion over ten years, with USD 5.6 billion accounting for direct medical expenditures alone [10]. International evidence likewise reinforces the cost-saving potential of SSB taxation. Emmet et al. estimated that, from a healthcare system perspective, €2262 million could be saved with a 20% SSB tax in Germany [38]. Likewise, in Canada, a CAD 0.015-per-ounce tax on SSBs was projected to generate healthcare savings of CAD 37.5 billion [39]. While multiple studies highlight substantial reductions in healthcare costs following SSB taxation, methodological heterogeneity across economic evaluations limits direct comparability. Moreover, multi-sectoral actions are essential to support the long-term sustainability and effectiveness of SSB tax implementation, including educational campaigns and health-promotion strategies, as well as encouragement for industry reformulation of SSB products [41,42].

Although the healthcare savings estimated for the Brazilian health system may seem modest, it is essential to note that the cost reduction only applies to the public sector. Healthcare savings in the Brazilian public sector may represent a substantial reduction in government expenditures in the health system, considering that the Brazilian Unified Health System (SUS) covers approximately 75% to 80% of the population (~150 to ~160 million individuals). Furthermore, government expenditure within the SUS corresponds to lower costs per person than those of other health systems due to the economies of scale granted by universal coverage, particularly in comparison to countries relying on private healthcare coverage [43]. The Organization for Economic Co-operation and Development (OECD) estimates that 22% of Brazilian healthcare costs are spent on inpatient admissions, while the SUS accounts for 45% of Brazilian healthcare spending [44]. This means that the costs we were able to include here account for only a small proportion of the total disease burden modeled, and cost savings are potentially much greater. Discount rates of 5% are also higher than those used in many other countries. Finally, the included diseases account for 65% of deaths and 75% of DALYs attributable to BMI. Therefore, alongside the costs of HHD being excluded from this analysis, the total costs and health benefits of the tax are likely to be greater in practice.

Our current projections for T2DM and stroke are largely consistent with previous national modeling efforts. A previous study estimated a similar reduction in T2DM prevalence, projecting 1.2 million fewer cases after 20 years of a 20% tax on SSBs [9]. Higher effects were estimated in our model for IHD, particularly driven by differences in the estimated impact among men. These differences can be attributed to the updated prevalence estimates from the GBD 2021 [22] dataset. Regarding stroke, our estimate is consistent with the number of averted cases from a recent modeling study using a similar framework [7].

Although less commonly included in simulation studies of SSB taxation [34], we incorporated chronic back pain and hip and knee osteoarthritis as outcomes due to their substantial burden attributable to high BMI. These conditions carry a high burden of DALYs, both in Brazil [22] and in specific high-risk groups, as observed in studies focusing on adolescents and young adults [45] and women of childbearing age [46]. Mechanical stress and inflammation associated with excess weight are primary drivers of these musculoskeletal morbidities. Consequently, the projected decrease in SSB consumption contributes to a reduction in the prevalence of these cases, thus improving the overall health status of the population and reducing the associated healthcare costs [47,48].

Modeling studies in the literature evaluating the impact of SSB taxation across multiple health outcomes have similarly reported consistent population-level health benefits [41,42]. However, comparing absolute figures across studies is often challenging due to methodological heterogeneity. These differences include variations in beverage consumption data, definitions of taxable beverages, countries’ economic classifications, the theoretical modeling frameworks adopted, population size, and assumptions regarding cross- and own-price elasticities [10,34,37,49,50,51,52,53]. Instead, as a tentative step to facilitate future comparisons, we aimed to minimize these differences by presenting our estimates as QALY per million adults, using PPP-adjusted values, and reporting cases averted per 100,000 adults. This approach allowed us to focus on the overall direction and relative magnitude of the health impact of a 20% excise SSB tax on the seven selected NCDs, rather than on absolute numerical differences.

The analysis by income levels in our study reveals an important divergence from much of the international literature. While numerous modeling and quasi-experimental studies often demonstrate that SSB taxation yields the greatest health benefits in low-income populations, mainly due to their higher price elasticity and greater baseline consumption [37,54,55], our findings suggest the opposite pattern. We observed that the largest reductions in disease cases, the greatest QALY gains, and the highest healthcare cost savings were concentrated among individuals in the highest income groups. Although this result may appear counterintuitive, it aligns with recent national modeling work, which similarly projected that the most substantial benefits of SSB taxation in Brazil would accrue to high-income populations [10]. This divergence likely reflects context-specific factors, including differences in consumption patterns and substitution behaviors across income strata. Specifically, high-income individuals may be more effective at substituting SSBs with healthier, non-taxed alternatives (e.g., bottled water or unsweetened products) than low-income groups. For the latter, substitutions might include other energy-dense, unhealthy items, thereby muting the overall health benefit. This dynamic is consistent with equity-focused modeling, which indicates that taxation alone may be insufficient for low-income groups and that coupling SSB taxes with measures such as water subsidies is required to enhance health benefits for lower-income households [26]. A variety of effects of SSB taxes on inequalities have been found in previous research worldwide. In Indonesia, the same pattern was identified in this study, with higher-income groups benefiting more than lower-income groups [56]. Indeed, a more extreme increase in inequalities was observed, with the number of strokes prevented being 30 times greater in the highest versus the lowest income quintile. In an analysis of four European countries with SSB taxes, consumption inequalities remained unchanged in three and were modestly reduced in one [57]. In the UK, benefits accrued most in the lowest-income quintiles, with smaller benefits to the highest-income groups and a small harm to health in the middle quintile. It is apparent that baseline patterns of consumption, price sensitivity, and responsiveness to the health messaging element are potential causes of impacts on health inequalities.

Several limitations should be considered when interpreting the findings of this study, arising from both data constraints and the modeling framework’s structure. First, there are some limitations related to the demand estimation. The QAIDS method used to derive price elasticities does not capture purchases of foods and beverages consumed outside the home, which may lead to an underestimation of total SSB intake and, consequently, of the tax’s potential health effects [26]. In addition, the use of cross-sectional population surveys to assess consumption patterns and disease prevalence introduces inherent challenges: the design does not support longitudinal causal inference and may contribute to temporal bias or reverse causality. We assumed causality linking the SSB tax with reduced purchasing, consumption, and changes in BMI to health outcomes.

Second, the cost data available in the Brazilian context impose restrictions on the generalizability of the economic results. Cost estimates necessarily rely on information from the public health system, given the absence of comprehensive cost data for the private sector. Moreover, population survey respondents cannot be directly linked to individuals with hospitalizations recorded in the datasets, limiting the precision with which disease-specific costs can be attributed.

Finally, several established limitations of the PRIMEtime health modeling framework are relevant to the interpretation of our findings. Here, baseline BMI and SSB consumption are self-reported, so are likely to be underestimated. This would introduce a conservative bias into the results and avoid overstating potential impacts. Despite a strong consensus on the causal harms of elevated BMI, the magnitude of these effects is derived from observational studies, which raises the possibility of residual confounding. Moreover, the model relies on assumptions about future trends that are inherently uncertain. Specifically, we assumed that SSB consumption levels and the underlying trends in BMI and disease rates would remain stable over time. However, it is essential to note that our analysis employed the dynamic energy balance model proposed by Hall et al. (2011), which predicts how changes in energy balance affect body weight in adults [29]. The model takes into account that changes in body weight occur slowly after alterations in energy intake or expenditure, and considers the physiological adaptations that reduce energy expenditure as weight decreases. For this reason, the model can be used to predict the impact of public policies (such as taxing sugary drinks) on population weight, showing that small, sustained changes are needed to address the obesity epidemic.

Nevertheless, even with this more realistic representation of bodyweight dynamics, projecting far into the future introduces uncertainties in behavioral, metabolic, and epidemiological trends. We also excluded some relevant health outcomes here, such as dental caries or BMI-related cancers, so the estimates are very likely to be underestimated. The assumption of independence of disease outcomes may also confer a conservative bias, as any superadded impacts of multimorbidity on utility or healthcare costs were not accounted for.

Despite these limitations, the findings of this study indicate that implementing a 20% excise SSB tax in Brazil could yield substantial health gains while reducing long-term healthcare expenditure. These results provide timely evidence to inform an evolving fiscal policy context and represent a significant opportunity to translate modeling projections into concrete public health action. On 16 January 2025, Complementary Law No. 214/2025 was approved, establishing the core framework of Brazil’s consumption tax reform. This includes the dual value-added tax system (Tax on Goods and Services, IBS and Contribution on Goods and Services, CBS) and, notably, a selective excise tax (Imposto Seletivo, IS) on products deemed harmful to health or the environment, with SSBs explicitly included among the targeted categories. Although the specific SSB tax rate has not yet been defined (as of January 2026), implementation is expected to proceed in phases, with 2026 designated as an adaptation year, full tax collection beginning in 2027, and the transition to the new tax system anticipated to be completed by 2033 [58,59].

Furthermore, the present study contributes to the literature on the adoption of excise taxes to reduce inequalities in nutrition and health outcomes in developing countries. Previous studies in developed and developing countries have indicated substantial reductions in SSB consumption due to the impact of excise taxes on purchases [14,16,60,61,62]; however, there is a lack of studies estimating differences in health outcomes attributable to the impacts of excise taxation by income in developing countries. Evidence on the potential health impacts of taxation schemes according to income level in developed countries includes one study on the occurrence of caries and obesity among children and adolescents in England [63], one study on obesity-related cancers among adults in the United States [64], and one study on the lifetime occurrence of obesity-related diseases in the Australian population [65]. Findings from studies in developed countries indicated greater health benefits for low-income individuals [63,64,65]. However, an Australian study showed that low-income individuals would be responsible for paying a higher proportion of SSB taxes [65]. Nevertheless, the methods for the calculation of health outcomes vary substantially across studies, including target population and health conditions included in the estimates, in addition to the measurement of health outcomes, e.g., body mass index, dental caries, and quality-adjusted life years among children and adolescents in England [63]; incidence and mortality in the United States [64]; and health-adjusted life years and healthcare costs in Australia [65].

Our study highlights that high-income individuals in Brazil would accrue greater health benefits, considering incidence, prevalence, mortality, and lifetime healthcare costs attributable to obesity-related conditions, linked to reductions in SSB consumption due to taxation. Given the baseline parameters of the Brazilian population, with a higher prevalence of obesity among high-income individuals and the narrow differences in SSB consumption and their respective changes due to taxation across income levels, gains in health outcomes attributable to SSB taxes will likely favor richer individuals in Brazil. The evidence provided in the study may guide public policy decision-making processes throughout the design of the excise SSB tax in Brazil, which should occur between 2026 and 2027 [58,59]. Furthermore, the study contributes to fill the gap in the literature regarding health benefits attributable to SSB taxation in developing countries marked by high socioeconomic inequalities like Brazil, showing that the adoption of fiscal measures for health promotion should be connected to structural and contextual features, therefore escaping the “one size fits all” approach that may compromise public policy design, implementation, and outcomes [66].

International experience suggests that SSB taxation can influence consumption through multiple mechanisms, including anticipatory behavioral responses triggered prior to enforcement. In the United Kingdom, for example, manufacturers reformulated their products, and consumers reduced their purchases of SSBs shortly after the 2016 announcement, well before the 2018 implementation, illustrating a notable “announcement effect” [61,62]. In Latin America, however, evidence of such pre-implementation shifts remains limited. In Mexico [14,60] and Chile [16], significant reductions in SSB purchases were documented primarily after-tax enforcement and corresponding price increases, with no robust evidence of anticipatory behavioral change.

Our simulation results focus on universal interventions because they are a central priority in health policy. Like many countries, Brazil faces the challenge of designing policies that are both universal and proportionate to address health and social inequalities. In highly unequal contexts such as Brazil, combining universal measures with proportionate actions is essential. Universal interventions are a necessary first step, but on their own, they may inadvertently widen inequalities if not accompanied by targeted, proportionate strategies.

The present study emphasizes the need for compensatory measures to address the potential increase in health inequalities in the Brazilian population due to the excise SSB tax. The current design of the tax reform already includes tax refunds for low-income individuals, as reflected in IBS and CBS; however, additional mechanisms to foster health promotion strategies may be key to further improve measures tackling health inequalities in the country. The use of tax revenues from IS into investments in primary healthcare activities focusing on lifestyle changes and preventive actions could constitute an important initiative for advancing equity in the context of public health.

## 5. Conclusions

As this study projects, the forthcoming implementation of the tax presents an opportunity to generate population health gains and reduce healthcare expenditure, provided that the final tax design includes a sufficiently high rate, broad product coverage, and complementary public health measures. These measures should include public awareness campaigns, improved availability and affordability of healthy untaxed alternatives (such as water), and systematic monitoring of consumer substitution patterns. This comprehensive approach would maximize the likelihood of achieving the health and economic benefits estimated in this and previous modeling studies. Future studies, including younger age groups, may help assess the long-term impacts and evaluate the potential for reducing consumption early in life.

## Figures and Tables

**Table 1:** Change in energy intake (kcal/person/day) after 20% tax for the total population and income levels.

	Mean (95% CI) Change Energy Intake in kcal/person/day		
	All	Men	Women
Total population	−26.4 (−27.2; −25.5)	−28.5 (−29.7; −27.4)	−24.5 (−25.3; −23.6)
Lower Income level	−18.1 (−19.4; −16.8)	−17.5 (−19.2; −15.9)	−18.6 (−20.2; −17.1)
Upper Income level	−29.0 (−30.5; −27.4)	−31.9 (−34.0; −29.8)	−26.3 (−27.9; −24.6)

CI: Confidence interval.

**Table 2:** Prevalence (95% CI) of overweight and obesity at baseline and after a 20% excise sugar-sweetened beverage (SSB) tax.

	Men		Women	
	Baseline (%)	After 20% Excise SSB Tax (%)	Baseline (%)	After 20% Excise SSB Tax (%)
All Overweight	41.0 (40.1; 41.8)	40.0 (39.2; 40.9)	34.3 (33.5; 35.0)	33.7 (33.0; 34.5)
Age group				
20–24	29.9 (26.9; 33.0)	27.1 (24.2; 30.0)	26.6 (23.8; 29.3)	25.5 (22.7; 28.2)
25–29	38.9 (36.1; 41.7)	37.7 (34.9; 40.5)	29.4 (26.7; 32.0)	29.1 (26.4; 31.8)
30–34	41.5 (38.7; 44.2)	40.7 (37.9; 43.4)	30.4 (28.1; 32.6)	29.9 (27.6; 32.1)
35–39	43.6 (41.1; 46.1)	42.8 (40.4; 45.3)	35.4 (33.0; 37.7)	33.8 (31.5; 36.1)
40–44	45.1 (42.4; 47.7)	44.5 (41.9; 47.1)	36.3 (34.0; 38.6)	35.5 (33.3; 37.8)
45–49	44.5 (41.6; 47.3)	44.9 (42.0; 47.9)	36.6 (33.9; 39.4)	36.3 (33.6; 39.1)
50–54	42.8 (39.7; 45.9)	41.8 (38.8; 44.8)	37.2 (34.7; 39.7)	36.5 (34.0; 39.0)
55–59	42.3 (39.3; 45.2)	42.3 (39.4; 45.2)	35.8 (33.4; 38.2)	35.6 (33.2; 38.0)
60–64	43.4 (40.6; 46.2)	43.6 (40.8; 46.4)	38.7 (36.0; 41.3)	37.6 (34.9; 40.2)
65–69	42.9 (39.8; 46.0)	42.5 (39.4; 45.7)	38.1 (35.3; 41.0)	38.0 (35.2; 40.9)
70–74	39.2 (35.6; 42.8)	38.7 (35.2; 42.3)	36.0 (32.6; 39.3)	36.3 (33.0; 39.7)
75–79	38.1 (33.7; 42.6)	35.7 (31.5; 39.9)	33.9 (30.2; 37.6)	32.9 (29.3; 36.6)
80–84	41.1 (35.4; 46.8)	39.2 (33.6; 44.8)	33.1 (28.6; 37.7)	32.3 (27.8; 36.9)
85–89	35.1 (26.1; 44.1)	32.6 (23.7; 41.6)	31.4 (24.5; 38.3)	28.5 (21.6; 35.3)
90–94	32.3 (17.5; 47.2)	29.3 (14.6; 44.0)	25.0 (16.7; 33.3)	23.5 (15.2; 31.7)
All Obese	19.8 (19.1; 20.6)	18.2 (17.4; 18.9)	23.6 (22.9; 24.3)	22.1 (21.4; 22.8)
Age group				
20–24	9.4 (7.6; 11.3)	8.8 (7.0; 10.7)	13.5 (11.2; 15.8)	12.8 (10.6; 15.1)
25–29	15.8 (13.8; 17.8)	14.0 (12.1; 15.9)	19.7 (17.0; 22.4)	17.7 (15.1; 20.3)
30–34	22.4 (19.7; 25.1)	20.6 (18.0; 23.2)	23.6 (21.2; 26.1)	22.1 (19.7; 24.4)
35–39	24.6 (22.2; 27.0)	22.7 (20.4; 25.1)	25.0 (22.8; 27.3)	23.8 (21.6; 26.1)
40–44	22.8 (20.6; 25.0)	20.9 (18.8; 23.1)	27.0 (24.8; 29.2)	25.1 (23.0; 27.3)
45–49	24.6 (21.8; 27.4)	22.2 (19.6; 24.8)	25.8 (23.3; 28.3)	24.9 (22.4; 27.4)
50–54	22.8 (20.3; 25.3)	21.4 (19.0; 23.9)	26.7 (24.2; 29.1)	24.9 (22.5; 27.4)
55–59	22.3 (19.5; 25.1)	20.4 (17.6; 23.1)	26.8 (24.5; 29.1)	24.9 (22.7; 27.2)
60–64	21.1 (18.6; 23.5)	18.8 (16.5; 21.1)	25.2 (23.0; 27.4)	24.1 (21.9; 26.3)
65–69	17.8 (15.5; 20.1)	16.5 (14.3; 18.8)	26.2 (23.4; 29.1)	24.6 (21.8; 27.4)
70–74	17.8 (14.6; 21.1)	15.8 (12.6; 19.0)	25.2 (22.1; 28.3)	23.0 (20.0; 26.0)
75–79	15.0 (11.7; 18.3)	13.1 (10.0; 16.3)	19.8 (16.6; 22.9)	19.1 (16.0; 22.2)
80–84	11.4 (7.6; 15.3)	10.0 (6.2; 13.7)	18.9 (14.3; 23.5)	17.5 (13.0; 22.0)
85–89	4.3 (1.8; 6.9)	4.0 (1.5; 6.5)	14.8 (10.4; 19.1)	13.8 (9.5; 18.1)
90–94	10.9 (2.4; 19.5)	10.5 (2.0; 19.1)	12.5 (4.7; 20.3)	12.5 (4.7; 20.3)

CI: Confidence interval.

**Table 3:** Estimated impact of 20% excise sugar-sweetened beverage (SSB) tax on the number of cases averted per 100,000 adults, with 95% uncertainty intervals.

Disease	Number of Cases Averted per 100,000 Adults Mean (2.5th–97.5th Centile)	
Total population	Men	Women
Type 2 diabetes (T2DM)	−922 (−1017; −815)	−862 (−937; −781)
Ischemic heart disease (IHD)	−858 (−968; −752)	−212 (−269; −157)
Hypertensive heart disease (HHD)	−87 (−157; −14)	−401 (−553; −170)
Stroke	−117 (−158; −73)	−122 (−156; −87)
Knee osteoarthritis	−124 (−195; −56)	−206 (−307; −109)
Hip osteoarthritis	−34 (−51; −18)	−35 (−52; −18)
Low back pain	−672 (−935; −419)	−1424 (−1874; −982)
Lower income level		
Type 2 diabetes (T2DM)	−513 (−567; −450)	−477 (−517; −431)
Ischemic heart disease (IHD)	−677 (−737; −618)	−118 (−149; −87)
Hypertensive heart disease (HHD)	−49 (−90; −5)	−227 (−313; −95)
Stroke	−52 (−77; −29)	−69 (−88; −49)
Knee osteoarthritis	−63 (−102; −25)	−115 (−171; −62)
Hip osteoarthritis	−18 (−28; −9)	−20 (−30; −10)
Low back pain	−327 (−478; −179)	−813 (−1076; −555)
Upper income level		
Type 2 diabetes (T2DM)	−1049 (−1157; −919)	−975 (−1058; −878)
Ischemic heart disease (IHD)	−919 (−1045; −789)	−244 (−312; −177)
Hypertensive heart disease (HHD)	−109 (−192; −23)	−478 (−656; −214)
Stroke	−142 (−195; −91)	−142 (−184; −101)
Knee osteoarthritis	−147 (−233; −66)	−239 (−361; −125)
Hip osteoarthritis	−40 (−60; −21)	−41 (−62; −21)
Low back pain	−814 (−1118; −520)	−1678 (−2201; −1174)

**Table 4:** Summary outputs of sensitivity analyses for QALY gained (per million adults) and healthcare cost savings (PPP$, per adult) for modeled diseases * over the lifetime, by sex and income levels, with discount rates of 0% and 10%.

		Men	Women
		Mean (LCI; UCI)	Mean (LCI; UCI)
0% discount rate	Total population		
	Number of QALY gained	82,937	74,113
	(per million adults)	(73,610; 92,672)	(64,686; 82,352)
	Healthcare cost savings (PPP$)	−633,621	−913,063
	(per 100,000 adults)	(−743,536; −531,291)	(−1,113,245; −727,053)
	Lower income levels		
	Number of QALY gained	53,771	41,013
	(per million adults)	(47,790; 60,036)	(36,134; 45,886)
	Healthcare cost savings (PPP$)	−376,292	−529,446
	(per 100,000 adults)	(−438,872; −314,857)	(−657,439; −410,275)
	Upper income levels		
	Number of QALY gained	92,538	84,751
	(per million adults)	(82,085; 103,623)	(72,684; 94,348)
	Healthcare cost savings (PPP$)	−720,318	−1,040,104
	(per 100,000 adults)	(−848,197; −595,145)	(−1,282,944; −799,262)
10% discount rate	Total population		
	Number of QALY gained	6903	3783
	(per million adults)	(5658; 8107)	(3328; 4224)
	Healthcare cost savings (PPP$)	−156,013	−249,199
	(per 100,000 adults)	(−183,879; −130,342)	(−315,622; −187,481)
	Lower income levels		
	Number of QALY gained	5371	2121
	(per million adults)	(4255; 6551)	(1880; 2343)
	Healthcare cost savings (PPP$)	−105,003	−147,352
	(per 100,000 adults)	(−121,404; −89,560)	(−186,981; −111,899)
	Upper income levels		
	Number of QALY gained	7418	4335
	(per million adults)	(6117; 8842)	(3793; 4841)
	Healthcare cost savings (PPP$)	−174,704	−287,331
	(per 100,000 adults)	(−202,911; −142,640)	(−355,957; −211,213)

* Modeled diseases: Type 2 diabetes (T2DM), ischemic heart disease (IHD), hypertensive heart disease (IHD), stroke, knee osteoarthritis, hip osteoarthritis, low back pain. PPP: purchasing power parity.

## Data Availability

The datasets used and/or analyzed during the current study are publicly available. The datasets supporting the conclusions of this article are included within the article (and its additional files). Any additional information can be requested from the corresponding author.

## Supplementary Materials

The following supporting information can be downloaded at: https://www.mdpi.com/article/10.3390/nu18030435/s1, Supplementary File. Table S1. Parameters used in the model. Table S2. Price elasticity for selected beverages for the total, and the lowest and highest income populations. Table S3. Baseline body mass index (BMI). Table S4. Baseline beverage consumption. Table S5. Energy intake at baseline (kcal/day). Table S6. International Classification of Diseases (ICD) codes for selected non-communicable diseases. Table S7. Morbidity and/or Mortality relative risks for a 5 kg/m^2^ BMI increase. Table S8. Change in energy intake (kcal/person/day) after 20% tax for the total population and income levels. Figure S1. Deaths, prevalent cases, and incident cases of ischemic heart disease (IHD), by sex. GBD, 2021. Figure S2. Deaths, prevalent cases, and incident cases of stroke, by sex. GBD, 2021. Figure S3. Deaths, prevalent cases, and incident cases of type 2 diabetes mellitus (DM), by sex. GBD, 2021. Figure S4. Deaths and prevalent cases of hypertensive heart disease (HHD), by sex. GBD, 2021. Figure S5. Prevalent and incident cases of low back pain (LBP), by sex. GBD, 2021. Figure S6. Prevalent and incident cases of Osteoarthritis knee, by sex. GBD, 2021. Figure S7. Prevalent and incident cases of Osteoarthritis hip, by sex. GBD, 2021.

## Abbreviations

The following abbreviations are used in this manuscript:

BMI	Body Mass Index
BRL	Brazilian Reais
CAD	Canadian Dollar
CBS	Contribution on Goods and Services (Contribuição sobre Bens e Serviços)
CI	Confidence Interval
DALY	Disability-Adjusted Life Years
GBD	Global Burden of Disease
HHD	Hypertensive Heart Disease
IBS	Tax on Goods and Services (Imposto sobre Bens e Serviços)
ICD	International Classification of Diseases
IHD	Ischemic Heart Disease
IS	Selective Tax (Imposto Seletivo)
NCD	Noncommunicable Diseases
OECD	Organization For Economic Co-operation and Development
PNS	National Health Survey (Pesquisa Nacional de Saúde)
POF	Household Budget Survey
pp	Percentage Points
PPP	Purchasing Power Parity
PSUs	Primary Sampling Units
QALY	Quality-Adjusted Life-Year
QUAIDS	Quadratic Almost Ideal Demand System
SIH	Brazilian Public Hospitals Information System
SSBs	Sugar-Sweetened Beverages
SUS	Brazilian Unified Health System
T2DM	Type-2 Diabetes
UI	Uncertainty Interval
WHO	World Health Organization
