# Supplementary material for: Impact of Fiscal Policy for Sugar-Sweetened Beverages on Reducing the Burden of Disease and Healthcare Costs in Brazil: A Simulation Study
Source: Nutrients. 2026 Jan 28;18(3):435. doi: 10.3390/nu18030435 (PMC12899526; doi:10.3390/nu18030435)
Supplement: Supplementary file 1 [file nutrients-18-00435-s001.zip › nutrients-4079046-supplementary.pdf]

Supplementary File

Contents

Table S1. Parameters used in the model. 2
Table S2. Price elasticity for selected beverages for the total, and the lowest and highest income populations. 3
Table S3. Baseline body mass index (BMI). 4
Table S4. Baseline beverage consumption. 4
Table S5. Energy intake at baseline (kcal/day). 5
Table S6. International Classification of Diseases (ICD) codes for selected non-communicable diseases. 5
Table S7. Morbidity and/or Mortality relative risks for a 5 kg/m2 BMI increase. 6
Table S8. Change in energy intake (kcal/person/day) after 20% tax for the total population and income levels. 7
Figure S1. Deaths, prevalent cases, and incident cases of ischemic heart disease (IHD), by sex. GBD, 2021. 8
Figure S2. Deaths, prevalent cases, and incident cases of stroke, by sex. GBD, 2021. 9
Figure S3. Deaths, prevalent cases, and incident cases of type 2 diabetes mellitus (DM), by sex. GBD, 2021. 10
Figure S4. Deaths and prevalent cases of hypertensive heart disease (HHD), by sex. GBD, 2021. 11
Figure S5. Prevalent and incident cases of low back pain (LBP), by sex. GBD, 2021. 11
Figure S6. Prevalent and incident cases of Osteoarthritis knee, by sex. GBD, 2021. 12
Figure S7. Prevalent and incident cases of Osteoarthritis hip, by sex. GBD, 2021. 12
References 13

Table S1. Parameters used in the model.

Variables	Input estimates	Data Source
Tax	20%	
Pass on rate	100%	
Brazilian population	By age and sex	2022 Brazilian Census Population [1]
Cross- and own-price elasticities for beverage categories	Price elasticity of Marshallian demand and expenditure elasticity, the entire population, and the 25% lowest and highest income groups<br>**Table S2**	Dassow et al. 2024[2]
Body mass index (baseline)	By age and sex<br>**Table S3**	PNS 2019 [3]
Beverage consumption (baseline)	By age and sex, for the entire population, and the 25% lowest and highest income groups<br>**Table S4**	POF 2017-2018 [4]
Total energy intake (baseline)	By age and sex, for the entire population, and the 25% lowest and highest income<br>**Table S5**	POF 2017-2018 [4]
Direct medical costs	By age and sex for selected NCDs	SIH 2021 [5]
Prevalence, Incidence, and Mortality for selected diseases	By age and sex<br>**Figures S1-7**	GBD 2021 [6]

POF: Pesquisa de Orçamentos Familiares (Household Budget Survey)
PNS: Pesquisa Nacional de Saúde (National Health Survey)
SIH: Sistema de Informações Hospitalares (Brazilian Public Hospitals Information System)
NCDs: Non-communicable diseases

Table S2. Price elasticity for selected beverages for the total, and the lowest and highest income populations.

↓Δq_i	Δp_j→	Group of beverages						
**Group of beverages**	Estimates for	Sugary juices/drinks	Soft drinks	Sugary dairy drinks	Sweetened juices/drinks	Sweetened dairy drinks	Light/diet soft drinks	Sports drinks/ energy drinks
Sugary juices/drinks	Total population	-1.017	0.041	-0.029	-	-	-	-
	Lowest income	-1.182	-	-	-	-	0.025	-
	Highest income	-0.795	0.097	-0.103	-	-	-0.118	-
Soft drinks	Total population	0.028	-1.066	-	-	-	-	-
	Lowest income	-	-1.134	-	-	-	-0.074	-
	Highest income	0.070	-0.859	-	-	0.163	-	-
Sugary dairy drinks	Total population	-	-	-1.012	-	-	0.050	-
	Lowest income	-	-	-0.984	-	-	-	-
	Highest income	-0.113	-	-0.857	-	-	-	0.109
Sweetened juices/drinks	Total population	-	-	-	-1.002	-	-	-
	Lowest income	-	-	-	-1.061	-	-	-
	Highest income	-	-	-	-0.897	-	-0.175	-
Sweetened dairy drinks	Total population	-	-	-	-	-	-	-
	Lowest income	-	-	-	-1.982	5.934	-	-
	Highest income	-	-	-	-	-1.532	-	-
Light/diet soft drinks	Total population	-	-	-	-	-	-0.920	-
	Lowest income	0.032	-0.082	-	0.045	-	-0.613	-
	Highest income	-0.115	-	-	-0.181	-	-0.771	-
Sports drinks/energy drinks	Total population	-	-	-	-	-	-	-1.003
	Lowest income	-	-	-	-	-	-	-1.077
	Highest income	-	-	-	-	-	-	-0.997

Source: Dassow et al. 2024 [2].

Table S3. Baseline body mass index (BMI).

	BMI (kg/m^2)			
	Men (n=40676)		Women (n=45149)	
Age group (years)	n	Mean (95%CI)	n	Mean (95% CI)
20-24	2878	24.58 (24.31;24.84)	3077	24.78 (24.46;25.11)
25-29	3457	26.05 (25.79;26.31)	3593	25.94 (25.60;26.27)
30-34	4149	27.02 (26.75;27.29)	4411	26.63 (26.27;26.99)
35-39	4647	27.39 (27.15;27.64)	4669	27.07 (26.82;27.32)
40-44	4088	27.22 (27.00;27.45)	4484	27.40 (27.15;27.66)
45-49	3870	27.38 (27.10;27.66)	4121	27.33 (26.97;27.70)
50-54	3738	27.20 (26.94;27.45)	4147	27.40 (27.13;27.67)
55-59	3656	26.92 (26.65;27.20)	4113	27.30 (27.05;27.55)
60-64	3201	26.79 (26.57;27.02)	3601	27.22 (26.96;27.49)
65-69	2601	26.46 (26.21;26.71)	3152	27.26 (26.95;27.57)
70-74	1878	26.33 (25.98;26.69)	2333	27.03 (26.66;27.39)
75-79	1300	25.65 (25.26;26.05)	1645	26.11 (25.70;26.52)
80-84	736	25.42 (24.92;25.92)	1025	25.76 (25.20;26.32)
85-89	321	24.39 (23.83;24.95)	498	24.96 (24.21;25.71)
90-94	114	24.93 (23.76;26.10)	217	23.97 (22.85;25.08)
95-99	31	25.64 (23.83;27.46)	51	-

CI: Confidence interval

Table S4. Baseline beverage consumption.

	Mean SSB consumption (ml/day)					
	Men			Women		
Age group (years)	Entire population	Lower income	Higher income	Entire population	Lower income	Higher income
20-24	443.1	245.9	598.7	375.4	329.0	425.1
25-29	420.4	260.8	531.7	338.0	248.3	389.5
30-34	408.9	281.6	481.8	323.1	235.2	375.5
35-39	363.1	194.8	462.3	316.2	229.3	334.8
40-44	362.6	195.2	496.6	286.8	185.1	299.0
45-49	321.9	198.2	433.7	257.2	196.4	286.6
50-54	290.6	170.8	328.7	290.6	185.0	330.4
55-59	304.0	141.0	370.2	280.3	179.0	344.4
60-64	300.7	148.6	335.9	297.2	173.8	344.9
65-69	283.1	207.7	349.0	279.8	132.9	326.1
70-74	313.4	270.2	349.3	296.1	278.9	361.7
75-79	353.5	160.9	409.2	301.6	199.8	319.3
80-84	319.3	243.8	424.2	312.6	178.2	372.9
85-89	416.4	331.6	491.1	350.7	185.4	367.7
90-94	317.0	168.0	361.4	297.8	94.2	210.8
95-99	307.8	278.1	375.3	155.7	-	599.7

SSB: sugar-sweetened beverages

Table S5. Energy intake at baseline (kcal/day).

	Mean Energy Intake (kcal/day)					
	Men			Women		
Age group (years)	Entire population	Lower income	Higher income	Entire population	Lower income	Higher income
20-24	3687.3	3543.1	3837.0	3018.6	2868.3	3002.2
25-29	3759.5	3701.4	3856.9	3088.9	3198.8	2902.5
30-34	3810.9	3608.4	3726.9	2950.6	3057.9	2819.7
35-39	3653.2	3467.9	3657.6	2786.5	2822.5	2724.6
40-44	3591.1	3150.6	3907.2	2818.8	2912.1	2681.1
45-49	3534.6	3349.9	3660.5	2748.3	2839.9	2596.6
50-54	3465.8	3276.7	3466.7	2748.7	2793.4	2506.8
55-59	3411.8	3136.7	3490.4	2687.3	2858.8	2418.6
60-64	3171.8	2990.0	3207.4	2636.1	2750.1	2336.8
65-69	3367.7	2969.7	3747.5	2694.7	2832.6	2333.3
70-74	3114.3	2836.8	3120.2	2571.6	2698.9	2417.9
75-79	3119.8	3194.0	3446.1	2553.6	2593.6	3112.5
80-84	2985.7	2580.5	3206.3	2483.2	2561.8	2416.9
85-89	2861.6	2600.4	3283.0	2561.4	2547.0	1846.4
90-94	2646.7	2450.6	2481.3	2516.5	2546.0	2024.1
95-99	3082.7	3594.5	2745.2	2574.1	1950.5	2736.7

Table S6. International Classification of Diseases (ICD) codes for selected non-communicable diseases.

Disease	ICD Code
Ischemic heart disease	I20, I21, I22, I23, I24, I25
Type 2 Diabetes Mellitus	E11, E12, E13, E14
Stroke	I64
Hypertensive heart disease	I10, I11, I12, I13, I14
Low Back Pain	M545
Hip osteoarthritis	M16
Knee osteoarthritis	M17

Table S7. Morbidity and/or Mortality relative risks for a 5 kg/m2 BMI increase.

	Morbidity and/or Mortality risk* for both sex			
Age group (years)	IHD	T2DM	Stroke	HHD
20-29	2.274	3.547	2.472	3.122
30-34	2.018	3.455	2.235	3.0
35-39	1.724	3.349	1.979	2.769
40-44	1.599	3.16	1.826	2.573
45-49	1.567	2.864	1.733	2.407
50-54	1.520	2.624	1.635	2.281
55-59	1.466	2.417	1.543	2.159
60-64	1.414	2.215	1.455	2.035
65-69	1.364	2.046	1.380	1.955
70-74	1.319	1.896	1.304	1.860
75-79	1.274	1.740	1.228	1.792
80+	1.170	1.461	1.068	1.697

	Morbidity risk*				
	Osteoarthritis hip		Osteoarthritis knee		LBP
Sex	Men	Women	Men	Women	Both
All ages	1.110	1.112	1.370	1.375	1.100

*Extracted from Global Burden Disease Study 2019 [7].

IHD: Ischemic heart disease

T2DM: Type 2 Diabetes Mellitus

HHD: Hypertensive heart disease

Table S8. Change in energy intake (kcal/person/day) after 20% tax for the total population and income levels.

	Mean (95% CI) change energy intake in kcal/person/day	
	Men	Women
**Total population - Overall**	**-28.5 (-29.7; -27.4)**	**-24.5 (-25.3; -23.6)**
Age groups		
20-24	-37.1 (-40.4; -33.8)	-31.5 (-34.4; -28.7)
25-29	-36.5 (-40.9; -32.0)	-29.8 (-32.5; -27.0)
30-34	-33.0 (-36.8; -29.1)	-26.4 (-29.5; -23.2)
35-39	-28.4 (-31.5; -25.4)	-24.7 (-27.1; -22.3)
40-44	-28.3 (-32.4; -24.2)	-22.6 (-24.9; -20.3)
45-49	-24.8 (-27.7; -22.0)	-20.4 (-22.3; -18.6)
50-54	-21.8 (-24.2; -19.5)	-22.2 (-24.8; -19.7)
55-59	-23.8 (-26.2; -21.3)	-22.0 (-24.3; -19.7)
60-64	-22.7 (-26.0; -19.3)	-22.3 (-24.9; -19.8)
65-69	-21.1 (-23.9; -18.2)	-21.1 (-24.7; -17.4)
70-74	-24.0 (-28.3; -19.8)	-22.0 (-25.0; -18.9)
75-79	-30.5 (-38.5; -22.4)	-22.7 (-26.8; -18.7)
80-84	-25.7 (-32.5; -18.9)	-25.8 (-32.3; -19.3)
85-89	-32.1 (-47.4; -16.7)	-27.7 (-33.8; -21.6)
90-94	-21.2 (-27.7; -14.6)	-23.8 (-32.7; -14.9)
95-99	-27.9 (-42.2; -13.7)	-19.5 (-48.4; 9.4)
**Lower Income level - Overall**	**-17.5 (-19.2; -15.9)**	**-18.6 (-20.2; -17.1)**
20-24	-20.0 (-23.8; -16.2)	-27.5 (-33.1; -21.9)
25-29	-23.2 (-29.9; -16.5)	-22.3 (-27.0; -17.5)
30-34	-23.3 (-29.7; -16.9)	-18.6 (-21.3; -16.0)
35-39	-15.7 (-18.2; -13.1)	-18.1 (-21.0; -15.3)
40-44	-14.7 (-18.2; -11.1)	-15.0 (-18.1; -12.0)
45-49	-15.2 (-18.7; -11.7)	-16.5 (-20.8; -12.2)
50-54	-13.6 (-17.2; -9.9)	-14.7 (-18.0; -11.4)
55-59	-10.7 (-14.4; -6.9)	-14.2 (-17.9; -10.6)
60-64	-10.6 (-14.1; -7.1)	-11.4 (-14.4; -8.5)
65-69	-13.0 (-17.7; -8.3)	-9.8 (-13.7; -6.0)
70-74	-20.0 (-32.3; -7.7)	-20.0 (-28.0; -12.1)
75-79	-9.2 (-15.5; -3.0)	-20.2 (-30.6; -9.7)
80-84	-14.1 (-24.9; -3.3)	-13.6 (-22.1; -5.1)
85-89	-23.0 (-35.3; -10.7)	-14.4 (-24.5; -4.4)
90-94	-12.0 (-22.9; -1.1)	-5.5 (-11.8; 0.9)
95-99	-18.6 (-55.3; 18.1)	a
**Upper Income level - Overall**	**-31.9 (-34.0; -29.8)**	**-26.3 (-27.9; -24.6)**
20-24	-44.5 (-50.2; -38.8)	-30.6 (-36.3; -25.0)
25-29	-44.8 (-54.7; -34.8)	-29.6 (-34.3; -24.8)
30-34	-33.9 (-38.9; -28.8)	-31.5 (-40.0; -23.1)
35-39	-30.7 (-35.7; -25.7)	-23.9 (-29.9; -17.9)
40-44	-36.4 (-48.6; -24.2)	-23.5 (-27.0; -20.0)
45-49	-30.3 (-35.8; -24.9)	-22.0 (-25.2; -18.7)
50-54	-21.8 (-24.8; -18.8)	-23.6 (-27.7; -19.5)
55-59	-27.5 (-31.3; -23.7)	-26.5 (-30.1; -23.0)
60-64	-24.5 (-29.6; -19.4)	-26.2 (-30.8; -21.6)
65-69	-25.4 (-30.3; -20.4)	-23.8 (-30.8; -16.8)
70-74	-27.8 (-33.5; -22.1)	-26.7 (-31.5; -22.0)
75-79	-39.0 (-55.2; -22.9)	-25.5 (-31.7; -19.4)
80-84	-35.2 (-46.7; -23.8)	-35.1 (-46.8; -23.3)
85-89	-38.7 (-53.6; -23.9)	-30.0 (-37.9; -22.2)
90-94	-22.0 (-31.2; -12.8)	-21.5 (-33.0; -10.0)
95-99	-26.9 (-34.0; -19.7)	-77.8 (-138.9; -16.7)

CI: Confidence interval   a: missing data

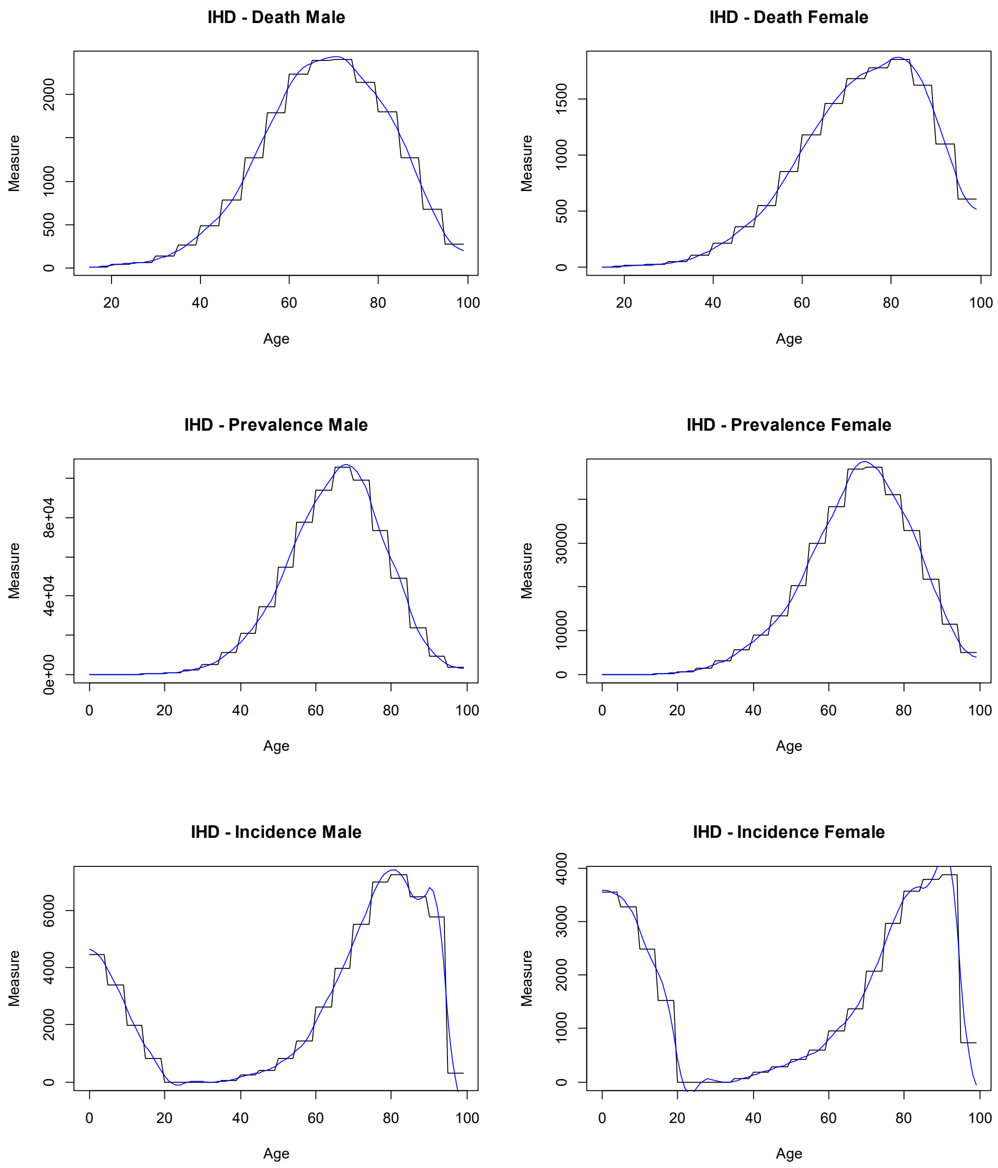

Figure S1. Deaths, prevalent cases, and incident cases of ischemic heart disease (IHD), by sex. GBD, 2021.

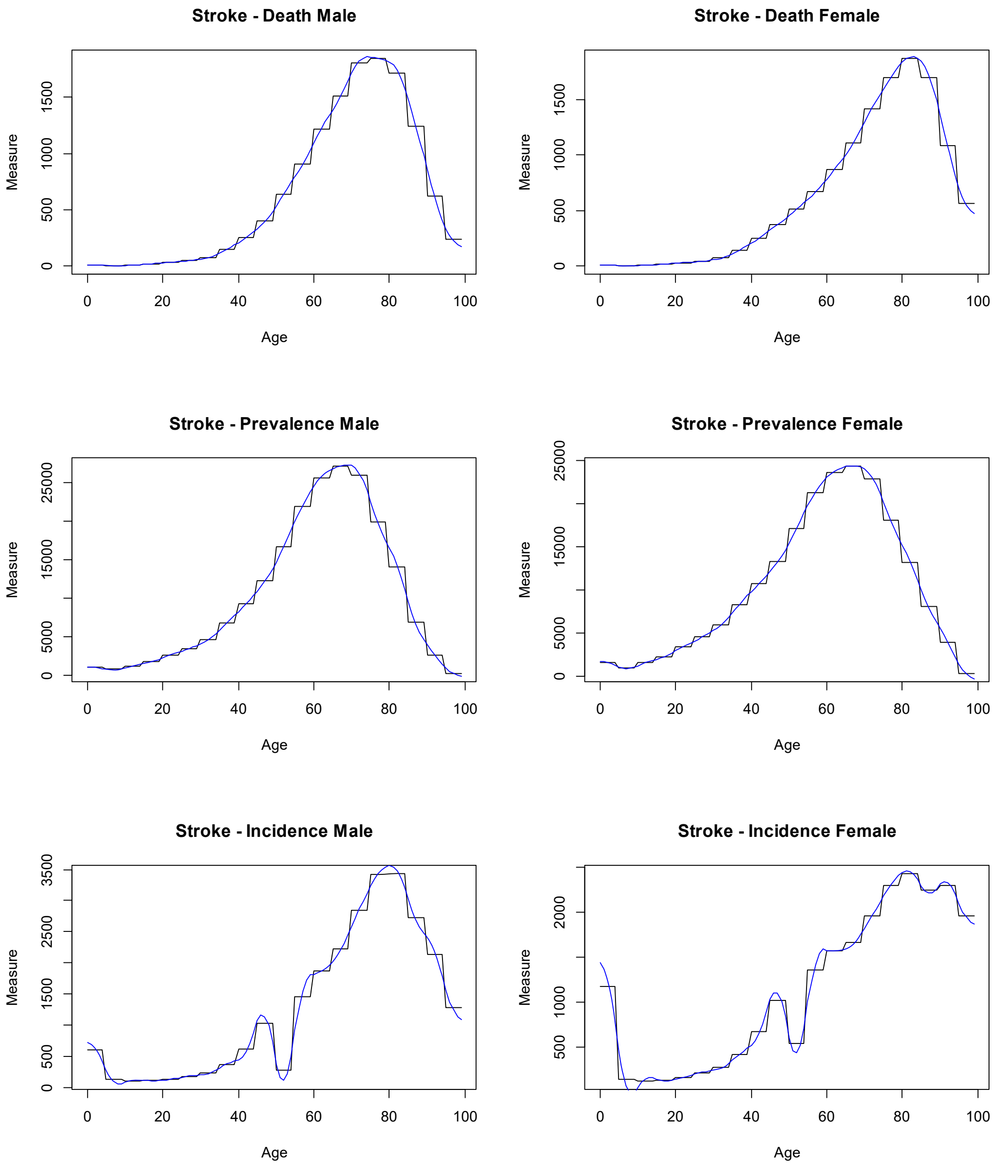

Figure S2. Deaths, prevalent cases, and incident cases of stroke, by sex. GBD, 2021.

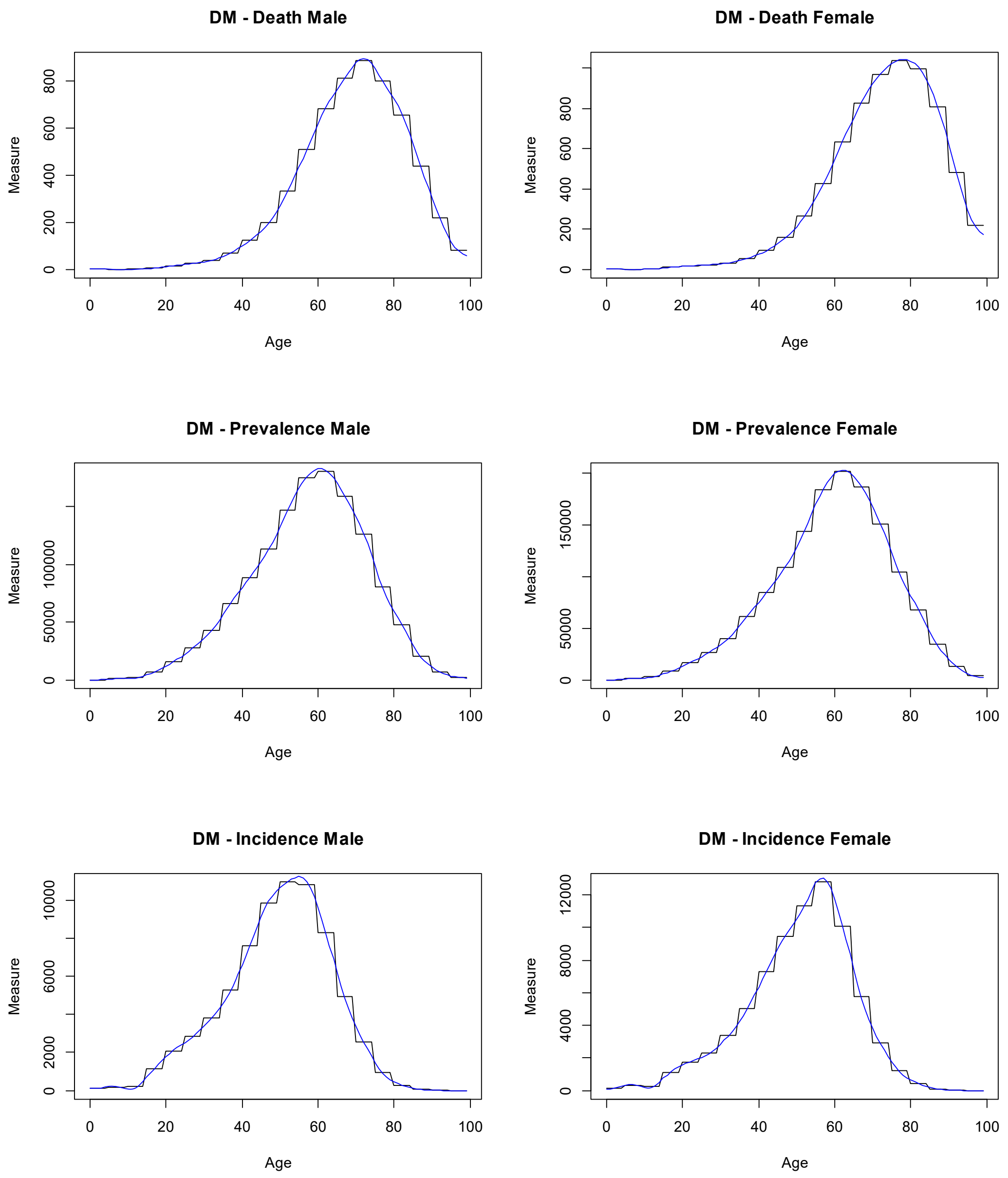

Figure S3. Deaths, prevalent cases, and incident cases of type 2 diabetes mellitus (DM), by sex. GBD, 2021.

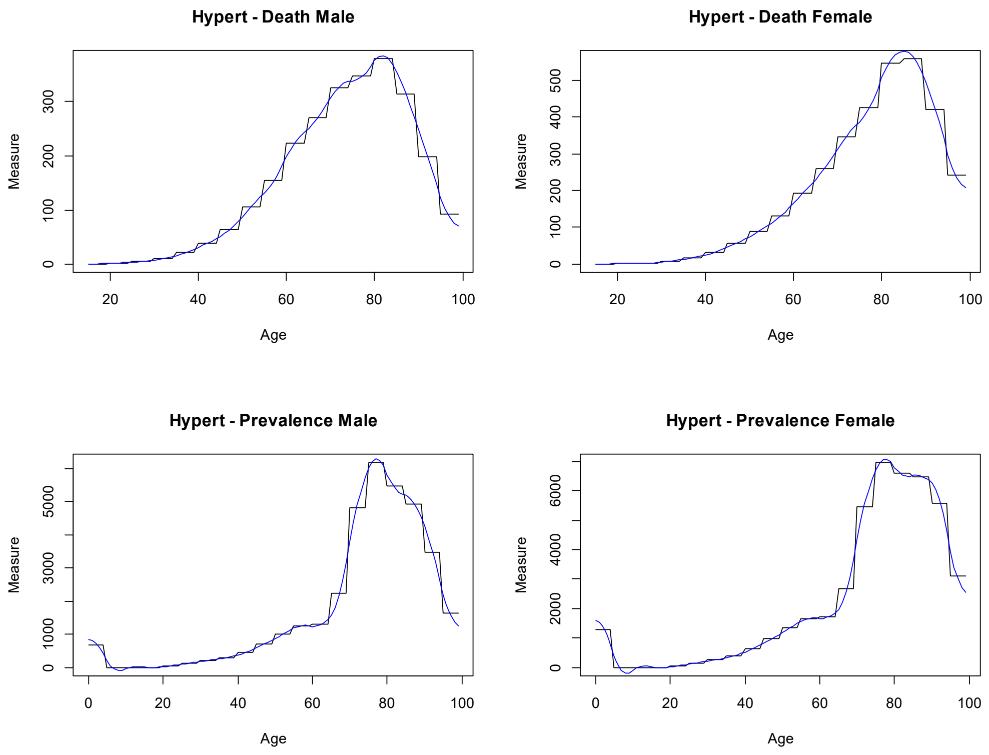

Figure S4. Deaths and prevalent cases of hypertensive heart disease (HHD), by sex. GBD, 2021.

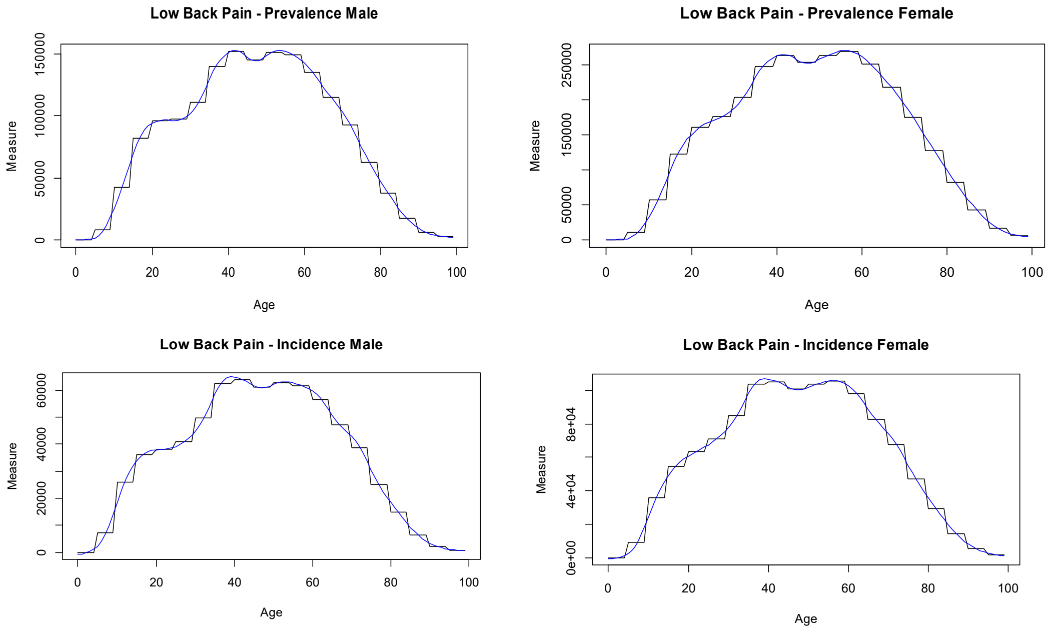

Figure S5. Prevalent and incident cases of low back pain (LBP), by sex. GBD, 2021.

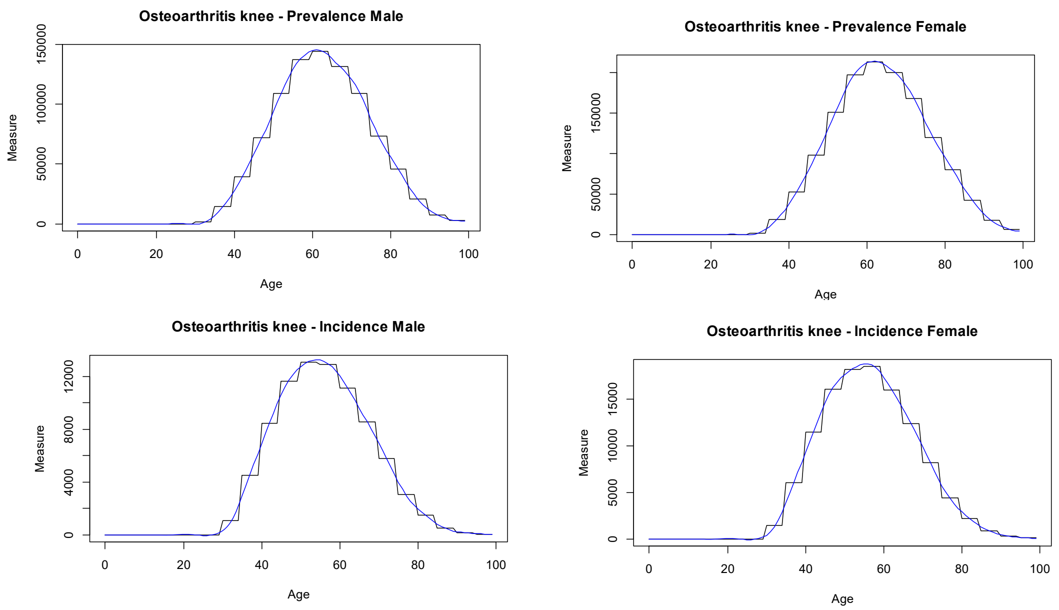

Figure S6. Prevalent and incident cases of Osteoarthritis knee, by sex. GBD, 2021.

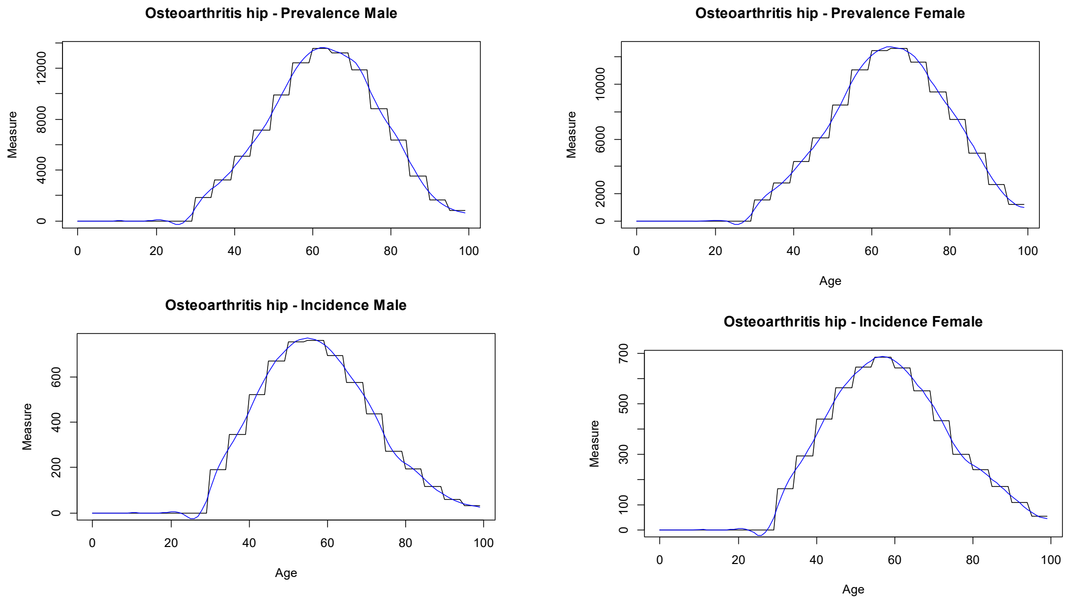

Figure S7. Prevalent and incident cases of Osteoarthritis hip, by sex. GBD, 2021.

References

[1] Instituto Brasileiro de Geografia e Estatística (IBGE). Censo Demográfico 2022. https://www.ibge.gov.br/estatisticas/sociais/populacao/22827-censo-demografico-2022.html?edicao=38166&t=resultados (accessed July 2, 2024).

[2] Dassow C, Almeida E. Tax on sugary drinks in Brazil: Simulation of impacts on the purchases of non-alcoholic drinks and family welfare. Food Policy 2024;128. https://doi.org/10.1016/j.foodpol.2024.102673.

[3] Instituto Brasileiro de Geografia e Estatística (IBGE). Pesquisa Nacional de Saúde 2019. Percepção do estado de saúde, estilos de vida, doenças crônicas e saúde bucal: Brasil e grandes regiões. Rio de Janeiro: 2020.

[4] Instituto Brasileiro de Geografia e Estatística (IBGE). Pesquisa de Orçamentos Familiares: 2017-2018: Análise do consumo alimentar pessoal no Brasil. Rio de Janeiro: 2020.

[5] Brasil, Ministério da Saúde. Transferência de Arquivos – DATASUS n.d. https://datasus.saude.gov.br/transferencia-de-arquivos/ (accessed September 26, 2024).

[6] Global Burden of Disease Collaborative Network. Global Burden of Disease Study 2021 (GBD 2021) Results. Seattle, United States: Institute for Health Metrics and Evaluation (IHME) 2022. http://ghdx.healthdata.org/gbd-results-tool (accessed April 22, 2025).

[7] Global Burden of Disease Collaborative Network. Global Burden of Disease Study 2019 (GBD 2019) Relative Risks. Institute for Health Metrics and Evaluation (IHME) 2020. https://ghdx.healthdata.org/record/ihme-data/gbd-2019-relative-risks (accessed June 25, 2025).
